# In-silico Prediction of Synergistic Anti-Cancer Drug Combinations Using Multi-omics Data

**DOI:** 10.1038/s41598-019-45236-6

**Published:** 2019-06-20

**Authors:** Remzi Celebi, Oliver Bear Don’t Walk, Rajiv Movva, Semih Alpsoy, Michel Dumontier

**Affiliations:** 1 Maastricht University, Institute of Data Science, Maastricht, Netherlands; 2 Columbia University, Department of Biomedical Informatics, New York City, USA; 3 Stanford University, Department of Genetics, Palo Alto, USA; 4 Turkish-German University, Department of Molecular Biotechnology, Istanbul, Turkey

**Keywords:** High-throughput screening, Computational models

## Abstract

Chemotherapy is a routine treatment approach for early-stage cancers, but the effectiveness of such treatments is often limited by drug resistance, toxicity, and tumor heterogeneity. Combination chemotherapy, in which two or more drugs are applied simultaneously, offers one promising approach to address these concerns, since two single-target drugs may synergize with one another through interconnected biological processes. However, the identification of effective dual therapies has been particularly challenging; because the search space is large, combination success rates are low. Here, we present our method for DREAM AstraZeneca-Sanger Drug Combination Prediction Challenge to predict synergistic drug combinations. Our approach involves using biologically relevant drug and cell line features with machine learning. Our machine learning model obtained the primary metric = 0.36 and the tie-breaker metric = 0.37 in the extension round of the challenge which was ranked in top 15 out of 76 submissions. Our approach also achieves a mean primary metric of 0.39 with ten repetitions of 10-fold cross-validation. Further, we analyzed our model’s predictions to better understand the molecular processes underlying synergy and discovered that key regulators of tumorigenesis such as TNFA and BRAF are often targets in synergistic interactions, while MYC is often duplicated. Through further analysis of our predictions, we were also ble to gain insight into mechanisms and potential biomarkers of synergistic drug pairs.

## Introduction

The last decade has seen a revolution in the discovery of small molecule cancer drugs^1,2^. Drug development has trended away from the one-drug-fits-all paradigm towards a diverse array of targeted agents that exploit specific knowledge of individual tumors^3^. While this approach can provide success, the confinement of drugs to a single target fails to take into account the complex etiologies of many cancers^4^. Specifically, the single target model is highly susceptible to the genetic diversity of tumors; one cell with a resistance-conferring mutation can cause complete evolution of the tumor in a few months^5^. Thus, under the current system of drug development, acquired resistance and intratumor heterogeneity will continue to hinder effective and permanent cancer treatment.

Theoretically, combination drug therapy can address many of the limitations that single target agents cannot. The underlying rationale is that drugs targeting different components of an interconnected network (either a single pathway or two related pathways) can more effectively suppress a certain biological process^6^. Several model studies have supported this hypothesis: Simultaneous drug treatments are far more robust to mutation, since two unlikely independent events must happen instead of one (*i.e*., *p*_1_ ≈ 10^−6^, so ${p}_{2}\approx {p}_{1}^{2}\approx {10}^{-12}$)^4^. Further, even in the presence of cross-resistance mutations, combination therapy still offers potential for treatment^7,8^.

However, tangible development of drug combinations has lagged behind theoretical discussion, primarily because identifying successful combinations is a difficult problem. More often than not, simultaneous administration results in no interaction between drugs and thus no net beneficial effect (termed *additivity*), or adverse interactions leading to decreased efficiency and possible toxicity (*antagonism*). *Synergistic* combinations are drugs that amplify each other’s activity, leading to elevated effects at low concentrations and, thus, reduced toxicity^9^. Picking out these synergistic combinations from the millions of possibilities requires meticulous experimentation and prohibitive levels of time and money^10^.

To aid in the identification and development of combination therapies, a few *in silico* methods have been proposed to predict successful drug pairs for further experimental tests in recent years. DrugComboRanker, the method by^11^, identifies synergistic drugs that target different signaling modules of a given disease network. However, this approach is limited to identification only of combinations that have known disease pathway interactions and is also highly susceptible to false positive pathway cross-talk. A method called DIGRE, proposed by^12^, works by identifying secondary drugs that are more effective on cells post-treatment with the first drug. However, DIGRE relies on knowledge of differentially expressed genes post-drug treatment, for which data is not widely available or practical to obtain in a clinical setting; perhaps because it considers synergy for sequential drug treatment, which has been shown to be ineffective at overcoming tumor resistance^7^. Another approach, RACS, identifies labelled drug combinations that are most similar to unlabelled combinations in the context of seven target-related features, and then incorporates overlap of differentially expressed gene signatures to predict synergy^13^. Like DIGRE, RACS also relies on elusive post-treatment data, but its feature set also limits its predictions to direct drug-protein interactions; our work on compensatory pathway analysis shows that these first-order synergistic effects are far from exhaustive. Huang *et al*.^14^ developed a computational model to predict drug combinations by using clinical side effects (SE) from post-marketing surveillance and the drug label. A database including 349 approved drug combinations was constructed with integration of drug information from SIDER, TWOSIDES, and DCDB sources. Logistic regression prediction model with 10-fold cross validation was utilized to determine predictive power of drug-drug combinations (DDC) relying on top 3 SE features identified by decision tree: pneumonia, haemorrhage rectum, and retinal bleeding. This approach does not use gene expression, pathway, and protein-domains information. They only look for marketed drugs in combination. Li *et al*.^15^ aimed to predict synergistic drug combinations with various features including drug chemical structure similarity, target distance in protein-protein network, and targeted pathway similarity. They also used fifteen pharmacogenomics features using drug treated gene expression profiles and built a prediction model for synergistic drug combination using the Random Forest method. They only used gene expression profile data of MCF7 cell line following drug treatment on the cell line from CMap. Zhao *et al*.^16^ developed a computational method for predicting synergistic activity of drugs used in combinations by integrating molecular and pharmacological data. They used STITCH, Drugbank and TTD databases to obtain compound-protein interactions. As a result of their analyses, they predicted 16 possible drug combinations. They reported that 11 out of their 16 predictions had already been identified as effective in the literature.

Predicting synergistic combinations using a wide range of cancer cell lines and drugs is much more challenging due to heterogeneity at molecular, chemical and biological level. Prior approaches have been limited by small dataset size and low data variety that could not reflect the extent of the standard prediction challenge. The DREAM AstraZeneca-Sanger Drug Combination Prediction Challenge offered one of the largest combinatorial cell line screening datasets, which also includes molecular data and chemical/biological data^17^. The dataset quantifies drug synergy with the Loewe model, defined as calculating the excess cell kill rate over the expected additive kill rate when the drug combination, is administered to cancer cell lines. The molecular information contained somatic mutations, copy-number alterations, DNA methylation, and gene expression profiles measured before drug treatment; and the compound information included putative drug targets, and where available, chemical properties. Here, we present our machine learning model developed to predict synergistic drug combinations for the DREAM AstraZeneca-Sanger Drug Combination Prediction Challenge. In order to best encapsulate the biological patterns underlying this synergy, we explored the most predictive and biologically relevant features for the prediction of drug synergies and trained a machine learning model using the features that characterise drugs and cell lines.

Our submission for Subchallenge 1 A of the DREAM AstraZeneca-Sanger Drug Combination Prediction Challenge was ranked in top 15 out of 76 submissions according to the primary metric used by the challenge organizers. Our machine learning model obtained the primary metric = 0.36 and the tie-breaker metric = 0.37 in the extension round of the challenge {https://www.synapse.org/#!Synapse:syn4231880/wiki/411305}. Our approach also achieves a mean primary metric of 0.39 with ten repetitions of 10-fold cross-validation. Through further analysis of our predictions, we were also able to gain insight into mechanisms and potential biomarkers of synergistic drug pairs. By automatically combining single-target drugs for synergistic therapy, our work paves the way towards efficient and widespread combinatorial cancer treatment.

## Results

### Model performance

#### Machine learning models accurately predict synergy

**Figure 1:**
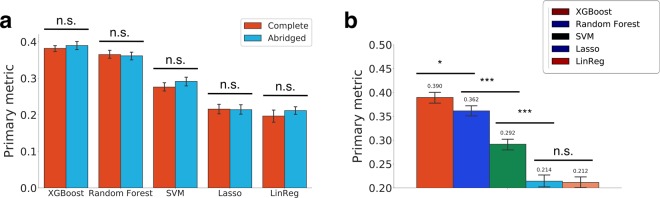
(**a**) Comparison of Primary metrics (weighted average Pearson correlations - WAPCCs) with the full and abridged feature sets. (**b**) Comparison of Primary metrics of the five models using the abridged feature set. *P < 0.01, ***P < 10^−4^, two-sample *z*-test. All error bars denote bootstrapped 95% confidence intervals. LinReg, Linear Regression; SVM, Support Vector Machine; n.s., not significant.

We began by comparing model performance training on the complete feature set (111,168 features) that contains all expression, copy number, and mutation data, and training on the abridged feature set (2121 features), to see if the latter completely encoded the relevant information. The abridged feature set includes the drug and cell line features that are biologically informative for drug synergy which have been extracted to train the machine learning models. Indeed, the abridged set performs equivalently for regression tasks across all five models (Fig. 1a), supporting our hypothesis that biologically informed feature curation can reduce overfitting and improve predictions. Thus, subsequent training was performed using the abridged set, both for its better performance and its faster training time.

Our next goal was to identify the most accurate model. We performed ten trials of 10-fold CV, and XGBoost and Random Forest stood out significantly from the others for regression (*P* < 5 × 10^−4^, two-sample *z*-test). With post-tuning, XGBoost achieved a weighted average Pearson correlation of *WAPCC* = 0.39; random forest was the next best model with *WAPCC* = 0.36 (Fig. 1b). Since XGboost was significantly better than Random Forest (*P* < 0.01, two-sample *z*-test), we used the XGBoost model for all downstream analyses.

#### Tuning XGBoost parameters

**Figure 2:**
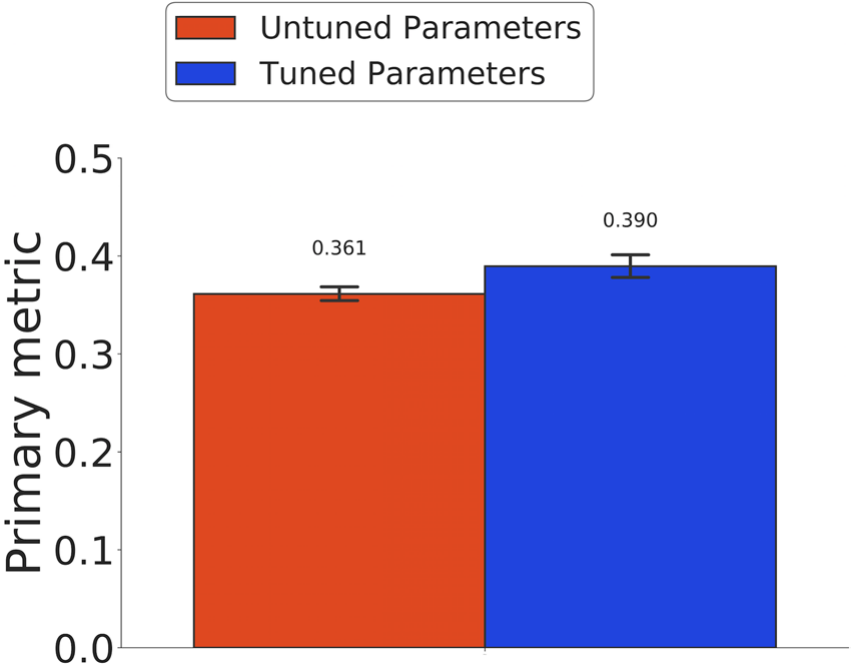
Parameter tuning of XGBoost. Comparison of ten repetitions of 10-fold cross validation weighted average Pearson correlations with the tuned parameters (*n*_*estimators* = 500, *max*_*depth* = 8, *subsample* = 0.75 and *colsample*_*bytree* = 1.0) and untuned default parameters (*n*_*estimators* = 250, *max*_*depth* = 8, *subsample* = 1.0 and *colsample*_*bytree* = 1.0) was obtained. Error bars denote bootstrapped 95% confidence intervals.

XGBoost performance and training time is heavily affected by choice of parameters^18^. We optimized four of these variables that cause most deviation: number of trees used (*n*_*estimators*), the maximum number of decisions (*max*_*depth*) for each tree, subsample ratio of observations and features (*subsample* and *colsample*_*bytree*) used to build each tree. Holding *n*_*estimators* constant, we varied the other three parameters and calculated cross-validation error at each step. We observed the minimum error with *max*_*depth* = 8. After setting the max depth to 8, we repeated the same process and varied the other three parameters. Error converged asymptotically for these iterations, so we took the best parameter values (*n*_*estimators* = 500, *max*_*depth* = 8, *subsample* = 0.75 and *colsample*_*bytree* = 1.0) that reached minimum error. Figure 2 shows the differences in regression performances (evaluated by ten trials of 10-fold CV) of the tuned vs. untuned models (*P* < 0.01).

### Biological interpretation

#### Feature importance analysis identifies biomarkers of synergy

**Figure 3:**
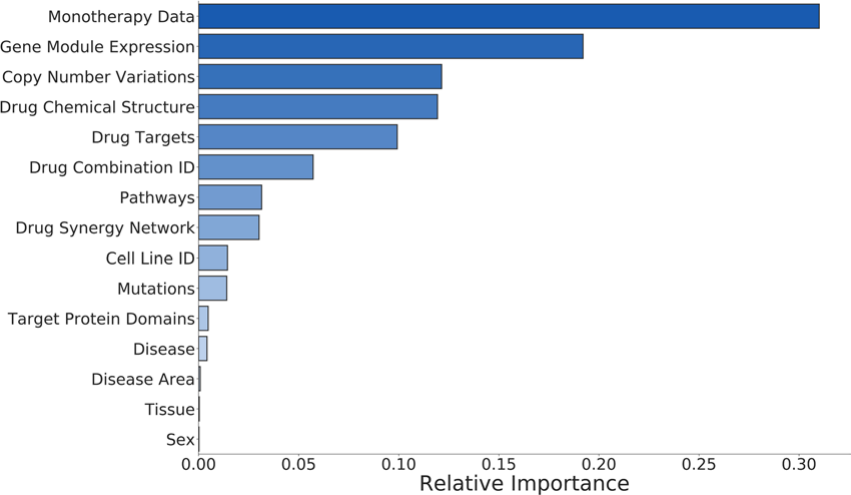
Bar plot of XGBoost feature group importances. For each training variable, an importance score is calculated as the improvement in predictive accuracy when that variable is included. Constituent scores (*e.g*., the 53 individual importance scores for gene expression modules) are summed to determine the net importance of each feature class. The *x*-axis units represent fractional contribution (sum of bar lengths = 1).

To determine biological factors underlying drug synergy, we computed an importance metric that represents percent contribution to the XGBoost model’s prediction for each of the 2121 features (computed as accuracy improvement when that feature is included). We first looked at the total group scores for the 15 types of features (Fig. 3). As expected, trivial information (drug combination ID, cell line ID, tissue, disease, and sex) did not contribute much (8% total), justifying our creation of a more sophisticated feature set. The monotherapy features are the most informative features which accounted for 31% of predictive power. Notably, genomic context (expression, CNV, mutations) accounted for 32% of predictive power. Our three novel drug synergy network features had a net score of 3%, indicating their promise for future research towards any type of drug interaction prediction. Target protein domains did not help much (0.5%), perhaps indicating that most drugs were not promiscuous (and thus, the putative targets alone held most relevant information).

**Figure 4:**
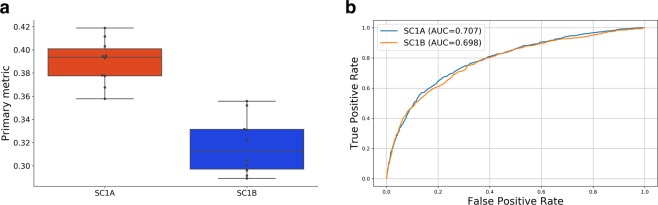
(**a**) Comparison of Primary metrics (weighted average Pearson correlations - WAPCCs) using all available molecular data (SC1A) and the molecular data excluding the monotherapy and gene expression feature sets (SC1B). (**b**) Comparison of the area under the receiver operating characteristic curve (ROC-AUC) with/without the monotherapy and gene expression feature sets.

Although monotherapy and gene module expression are the two most important feature sets, we looked at the performance of a model which excluded these data in order to mimic current clinical settings. Also the competition organizers divided the first challenge (SC1) into two parts; in SC1A the competitors were asked to make prediction using all available data, whereas in SC1B the use of molecular data was limited to mutation and copy number variation. Figure 4 shows differences between the XGBoost models trained with/without the monotherapy data and gene expression. We also plotted the area under the receiver operating characteristic curve (ROC-AUC) to evaluate the performance of the binary predictions. In generating ROCs, we trained the models for classification by binarizing the target values. The threshold was set at 20.0 as suggested by the challenge organizers (any score above 20.0 is represented by a 1, other scores map to 0). When gene expression and monotherapy information are excluded, the performance of XGBoost model drops significantly for real synergy value prediction (*WAPCC* = 0.32) but this difference is not significant in terms of binary predictions (*AUC* = 0.70).

Co-expression network created by the WGCNA approach identified cohesive modules having distinct gene expression patterns. Cohesiveness is a measure of how tightly a particular gene fits into its module. The more cohesive the module, the more similar the co-expression relationships are across the module. Module enrichment analysis performed after module identification showed that a majority of the modules have indeed biological functionality. When focused on these biologically important modules, the WGCNA detected several centrally located intramodular hub genes within the modules. These hub genes are highly connected to the rest of the genes in the module, so they can be regarded as major components of the module. Indeed, expression profiles of these hub genes are highly correlated to module eigengene values of the modules they belong to. In this respect, it seems that they are the most important genes in the modules, so effective drugs likely to attack to these hub genes. Thereby, they can be considered as biological targets or biomarker candidates for drug sensitivity.

We next conducted a finer analysis, looking at the most significant of the 2121 variables individually (using the same percent contribution importance metric) to extract more specific biological information regarding synergy. For drug targets, a master cancer signaling protein^19^, tumor necrosis factor alpha (TNFA), ranked highest. B-raf V600E, a mutant of the oncogene *BRAF* that determines drug sensitivity via signaling^20^, and ATR, a kinase protein regulating DNA repair^21^, were the next most significant. Using two-sample Kolmogorov-Smirnov test, TFNA (*P* < 2.3^−3^) and B-raf V600E (*P* < 2.0^−15^) can significantly favour synergistic combinations, while the drug combinations targeting AKT1 (*P* < 0.05) and ATR (*P* < 2.2^−14^) proteins are likely to be antagonistic. For copy number variation, repetitions or deletions in the *MYC* and *NFKBIA* genes were most significant. MYC is a transcription factor whose copy number has been strongly correlated to colon cancer in the past^22^, whereas NFKBIA is involved in several cancer pathways but only has a tenuous link between CNV and cancer^23^. Pathway analysis revealed that cell differentiation, apoptosis, and cancer signaling processes were most important. The membrane active transport pathway also ranked highly, perhaps for its role in regulating drug influx and efflux^24^. We also analyzed potential synergy mechanisms of highly ranked mutations, summarized in Table 1. Five of these mutations have been previously shown to be cancer risk factors. Thus, feature importance analysis combined with results from existing literature implicates the aforementioned variables as novel potential biomarkers of synergistic drug effects.

**Table 1:** Most predictive mutations of synergy, identified by XGBoost. The location of the mutation is given as its chromosome followed by its genomic coordinate. Brief hypotheses for the influence of each mutation on synergy are proposed. Pos., Position; PPI, Protein-Protein Interaction; MSM, Missense Mutation (results in different amino acid).

Mutation Pos.	Gene	Pathology	Hypothesized Influence on Synergy	References
3:179218294	*PIK3CA*	Breast	MSM in PIK3a domain changes drug sensitivity	^44^
11:36489991	*TRAF6*	Bladder	Affects MAPK apoptotic signaling pathway	^45^
9:130862983	*ABL1*	Colon	Confers resistance to tyrosine-kinase inhibitor drugs	^46^
4:102613584	*NFKB1*	Colon	MSM in binding domain affects transcription regulation	—
22:41166649	*EP300*	Lung	MSM disrupts transcriptional co-activation	—
10:121565526	*FGFR2*	Colon, Lung	Increased expression, affecting FGF signaling pathway	^47^
12:25245351	*KRAS*	Colon, Lung	Disrupts Akt/mTOR pathway through PPI networks	^48^

## Discussion

Cancers are complex diseases that are regulated by multiple complementary or redundant pathways. As a result, acquired drug resistance is an issue plaguing the vast majority of current single-agent chemotherapy regimens. The design and development of targeted drug combinations that disrupt multiple modes of metastasis is thus becoming increasingly necessary. Here, we establish the first comprehensive machine learning framework that successfully predicts synergistic drug combinations and presents opportunity for further exploratory biological analysis.

In this study, IC50 is used as a sensitivity measure for predicting drug synergy since GDSC and DREAM studies reports the sensitivity of all the screened anti-cancer drugs with IC50. This measure is not a powerful indicator of drug activity as IC50 could not be measured when maximum drug concentration is not sufficient for killing the cells/cell lines. Indeed, we noticed that most of the screened cell lines in the GDSC and DREAM studies does not reach an IC50 point within screening concentration interval. In addition, we identified that there are substantial deviations in IC50 values reported for the cell lines screened by the same drugs in DREAM study. It shows either assay used in experimental procedure does not measure correct IC50 values or cell lines are genetically heterogenic, i.e, they consist resistant and sensitive sub-populations of cells. So using IC50 as a sensitivity measure might lead to underperformance of our *in-silico* models generated for predicting synergistic drug combinations. Instead of IC50, using alternative sensitivity measures such as Activity Area and Amax, which are regarded to be more reliable indicatiors of drug sensitivity, would improve the predictive power of our models and give us a more reliable picture of synergistic drug pairs.

This work takes a data-driven approach to drug synergy prediction, integrating comprehensive pharmacological data with molecular information to train powerful machine learning models. Importantly, we combine several different biological data types to build a comprehensive, novel feature set and thus optimize performance. We show that XGBoost is the most well-suited learning algorithm to synergy identification. Ultimately, our model’s high correlation, generalizability to external data, and *de novo* discovery of drug combinations currently undergoing clinical trials alongside novel synergistic pairs all support its predictive success over previous methods.

We provide the workflow that generates the feature set and the results so that other labs can easily use or extend this methodology. The workflow can also handle additional features or missing features and can be run on a standard desktop machine. Note that the performance and training time of the XGBoost model are greatly influenced by the hyperparameters that need to be tuned. The overall performance of the method may be improved with the addition of gene expression and monotherapy data, however such data are challenging to obtain in clinical settings owing to financial, logistic and technological reasons. We note some possible limitations in our model’s predictions. Using a synergy score as the output metric may not be ideal, since it is an integral over a wide range of concentrations (whereas in practice, treatments at lower concentrations are generally more clinically feasible). Additionally, computational models may report false positives, so our newly discovered combinations must be validated.

In the future, we hope to further explore undiscovered mechanisms of drug synergy. Specifically, drugs activating and repressing shared transcription factors via downstream effects has been recently suggested as a synergy mechanism^25^. We also plan to conduct experimental trials of predicted synergistic drug combinations on cancer-specific cell lines and patient organoid models to further support our *in silico* approach. Regardless, we expect our current framework to aid in rapid identification and development of synergistic drug combinations towards specific and comprehensive cancer treatment for all.

## Methods

**Figure 5:**
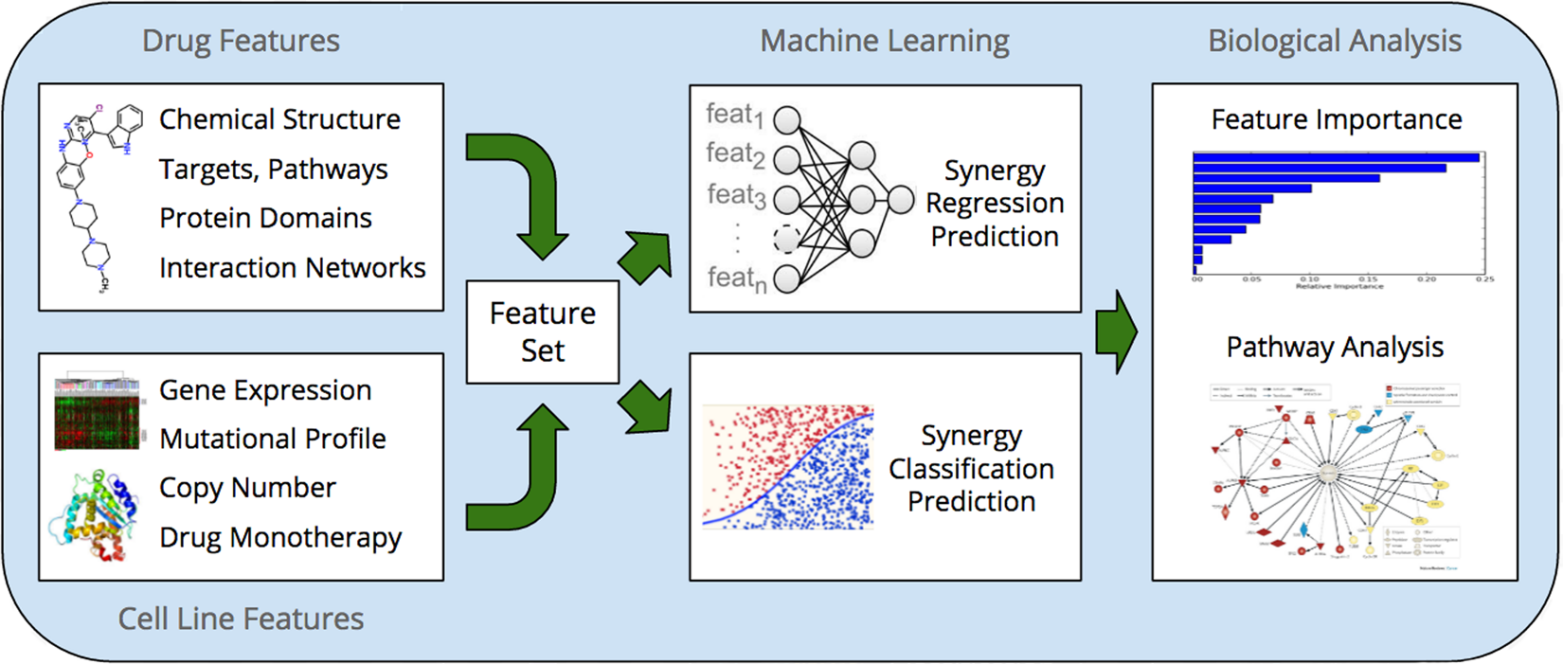
Our pipeline for modeling and analysis of drug synergy. We integrate features from two input streams: drug data and cell line data. We train our machine models on the compiled feature set and perform biological analysis of predictions to propose novel hypotheses explaining drug synergy.

To better understand drug-drug interactions and suggest viable synergistic pairs, we approached the problem by aggregating as much open data as possible to build an accurate predictive model. We subsequently analyzed our predictions to identify novel and plausible mechanistic synergy hypotheses. Our workflow spanned three stages: feature compilation, building and evaluation of machine learning models, and biological interpretation of our results (Fig. 5).

### Training data

We used a dataset recently released by AstraZeneca and the Dialogue for Reverse Engineering Assessments and Methods (DREAM) consortium^17^ as the core training data for our method. The data are composed of synergy scores for 2790 experiments (Subchallenge 1 Training set + leaderboard) across 167 drug combinations and 85 cell lines, representing a small fraction of the complete combinatorial space (2790/(167*85) = 19.6%) but still the largest combination drug screen to date. Synergy scores are defined by integrating experimental cell kill fraction minus the expected additive cell kill fraction as defined by the Loewe model^26^.

### Feature set

Pairs of drugs that are synergistic on one cell line are not necessarily synergistic on other cell lines^12^. Hence, we hypothesized that information on both the drugs and the tested cell line is predictive of synergy, making it necessary to incorporate both classes of features into our method. We extract the biologically relevant features, called abridged feature set. We detail the groups of features used to train our models below.

#### Chemical structure

Drug structure at the molecular level describes its binding activity. Chemical fingerprints are the most commonly used structural profile of drugs^27^. Fingerprints are bit vectors that indicate the presence (1) or absence (0) of various chemical features (*e.g*., a C=N group, a six member ring, *etc*.). To integrate fingerprints into our pipeline, we used the Python OpenBabel 2.3 library^28^ to take an input chemical formula (SMILES ID; given by AstraZeneca) and generate length 166 Molecular Access System (MACCS) binary structural feature lists^29^. For each drug combination, we used the sum of the two single drug bit vectors as features (*i.e*., a 2 represents both drugs having the feature, a 1 represents one of the drugs having the feature, and a 0 represents neither of the drugs having the feature; this mapping worked best) preserving similarity resolution across each of the 166 structural elements. While individual elements may not be relevant, we expect our model to learn combinations of structures that are predictive.

#### Drug targets

Targets can shed light on the biological processes that the drug controls. We started by using summed bit vectors of the putative targets (given as part of the AstraZeneca synergy dataset), of which there were 185 across all the drugs; thus, a 2 represents a shared target, a 1 represents a target of one the drugs, and a 0 represents a target for neither of the drugs. However, this matrix was sparse across the training dataset, since the drugs have a median of one putative target each.

#### Target protein domains

To account for other drug-protein interactions, we generated structural protein domain features for the targets of each drug and mapped them to drug combinations in the same 2, 1, or 0 format. We used four databases (Pfam^30^, Prosite^31^, SMART^32^, and SUPERFAMILY^33^ with 131, 97, 67, and 75 features, respectively) resulting in 370 total domain features. These features may account for cases in which drugs are not known to interact specifically with a given target, but they still have some binding affinity (termed a *promiscuous* interaction).

#### Targeted pathways

We also generated 309 features for the biological pathways involving the drug targets using the Kyoto Encyclopedia of Genes and Genomes (KEGG) database. The rationale for these features was to provide a direct read on the specific metabolic, signaling, and regulatory processes that the drug combinations disrupt, which may inform synergistic effects.

#### Drug synergy network

We have explored the network based features to see if drug synergy is transferable between the cell lines and distinguish synergistic drug combinations. Previous studies have reported predictive success using network topology of drug-drug interaction networks^13,34^. We built an undirected synergy network, in which two synergistic drugs are connected by an edge. We identified a drug combination as synergistic if the majority of synergy scores for that drug combination across cell lines is greater than 20. Using the synergy network, we extracted three features frequently used in social network link prediction for each drug pair: number of common neighbors, Jaccard coefficient, Adamic-Adar coefficient^35^.

The network proximity features for a drug pair (*x*, *y*) in the drug synergy network are defined as follows:

1 $${\bf{Common}}\,{\bf{Neighbors}}({\bf{x}},{\bf{y}})=|{\rm{\Gamma }}(x)\cap \,{\rm{\Gamma }}(y)|$$

2 $${\bf{Jaccard}}({\bf{x}},{\bf{y}})=\frac{|{\rm{\Gamma }}(x)\cap {\rm{\Gamma }}(y)|}{|{\rm{\Gamma }}(x)\cup {\rm{\Gamma }}(y)|}$$

3 $${\bf{Adamic}}/{\bf{Adar}}({\bf{x}},{\bf{y}})=\sum _{z\in {\rm{\Gamma }}(x)\cap {\rm{\Gamma }}(y)}\frac{1}{\mathrm{log}|{\rm{\Gamma }}(z)|}$$

where Γ(*x*) represents neighbors of node x, Γ(*y*) represents neighbors of node y in the drug synergy network.

#### Monotherapy information

To calculate synergy scores defined by excess over the Loewe additivity model, the AstraZeneca study also conducted cell viability assays for the 69 individual drugs involved in the 167 combinations^36^. These monotherapy features included, for each drug in the combination, the maximum concentration used in the assay, the IC50 value (concentration where half of maximum kill is achieved), the Hill coefficient *H* (slope of the dose-response curve), the max kill percentage *E*_inf_ and data quality check information.

#### Gene expression profiles using weighted correlation network analysis

**Figure 6:**
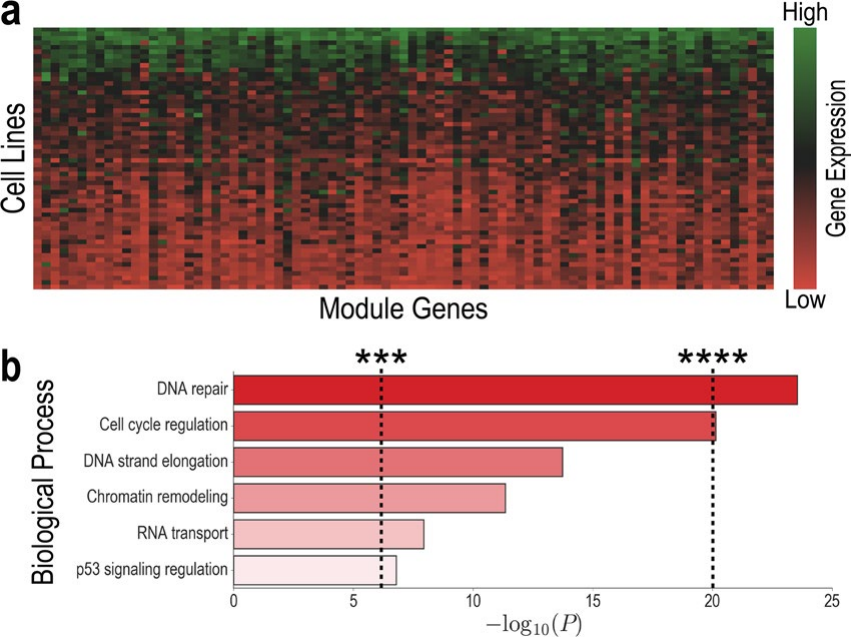
Weighted Gene Co-Expression Network Analysis identifies modules of correlated genes. (**a**) Expression heatmap for the 162 genes that form one of the modules. Note that in each cell line, the genes are either primarily highly expressed (green rows) or primarily lowly expressed (red rows), indicating that gene expression is correlated within modules. (**b**) Selected gene ontology biological term enrichments for genes in the cluster from (**a**) illustrate module-level biological function. ***P < 5 × 10^−7^, ****P < 10^−20^ (Hypergeometric test with Bonferroni multiple testing correction).

Microarray expression data of 17,419 genes were generated for the 85 cell lines by the Genomics for Drug Sensitivity in Cancer (GDSC) group^37^. However, using such a large number of gene expression values directly can be detrimental, since data for individual genes have noisy deviations across cell lines that are not biologically meaningful (we saw minimal improvement in model performance when we used raw expression). To overcome this issue and summarize biological processes that are otherwise difficult to learn, we leveraged Weighted Gene Co-Expression Network Analysis (WGCNA), a robust technique to identify systems-level gene modules^38^. Modules are determined by hierarchal clustering of the 17,419 × 17,419 gene expression correlation matrix^39^. As expected, genes within a given module have highly correlated expression profiles (Fig. 6a), but are also frequently enriched for Gene Ontology (GO) terms that indicate biological function (Fig. 6b). Thus, we used mean expression values of the 53 modules as cell line features.

#### Mutations and copy number variations

**Figure 7:**
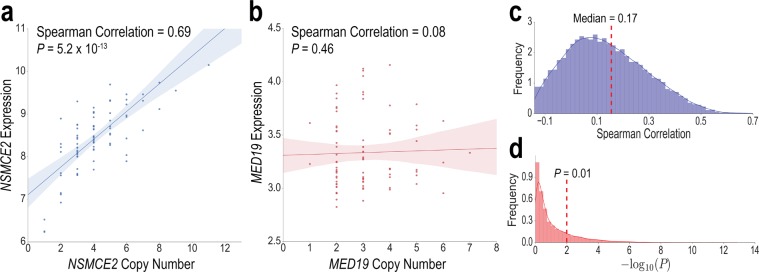
Expression and copy number variation (CNV) correlations differ across genes. (**a**) *NSMCE2* expression varies with CNV for the 85 cancer cell lines, while (**b**) *MED19* does not have a significant correlation. (**c**) Distribution (probability density) of Spearman rank correlations and (**d**) distribution of negative log *P*-values for all 17,419 genes. CNV of cancer genes with above median Spearman and a significant *P*-value were used as features. *P*-values are generated with Fisher’s *r*-to-*z* transformation for correlation testing.

Genomic sequence features also provided important information for cell line-specific context. The Catalogue Of Somatic Mutations In Cancer (COSMIC) database performed whole-exome sequencing of the 85 cell lines to identify coding single nucleotide polymorphisms (SNPs) and copy number variations^40^. In total, there were 75,281 SNPs that occurred in at least one cell line, but the vast majority of these mutations were not predictively relevant. We filtered out all SNPs in genes that are not in the KEGG cancer pathways, resulting in 876 features represented in binary format; these included *BRAF*, *TP53*, and other canonical tumorigenesis mutations^41^. Copy number variations (CNVs) are long, repeated segments of genes that have been increasingly implicated in disease in recent years^42^. To filter CNVs, we correlated the copy number of each gene with its expression across the 85 cell lines. Genes in the cancer pathways with a statistically significant, above median correlation (*P* < 0.01, Fisher’s correlation test; Spearman rank correlation >0.17) were hypothesized to possibly have functionally relevant CNVs (Fig. 7), and their copy numbers were included in the feature set (143 genes).

### Machine learning prediction

To learn patterns from the feature set and make accurate synergy score predictions, we trained multiple machine learning models with the AstraZeneca data to identify an optimal framework.

#### Types of models trained on the feature set

We trained five machine learning models on the feature set for synergy score prediction: linear regression, Lasso, support vector machine (SVM), random forest, and XGBoost. The first four models were trained using the {sklearn} Python package^43^, while XGBoost was trained using the {xgboost} Python package^18^. Linear regression fits a line to the training data by minimizing its *cost function*, which we chose to be the sum of the squared distances of the predictions from the actual synergy scores. Lasso is a linear regression with an L1-regularization term (*i.e*., the sum of the absolute values of the coefficients) in the cost function to prioritize models with smaller coefficients and thus reduce overfitting. SVM works by building a hyperplane in an *f*-dimensional feature space such that all dimensions are within *ε* of the target value and the L2-norm (sum of squared coefficients) is minimized. Random forest stochastically assigns a set of features and training examples to each of its *n* decision trees that are independently trained and then averaged for the final prediction. XGBoost is similar but adds L2-regularization and boosting, a method to prioritize the weights learned from the most mispredicted examples.

#### Evaluating predictive performance using cross validation

For evaluation of model performance using only the AstraZeneca dataset, we did ten repetitions of 10-fold cross-validation (CV). This involves randomly splitting the data into 10 equally sized segments, iteratively training on 9 of the 10 folds, and testing on the remaining tenth. We used the weighted average Pearson correlation coefficient (WAPCC) of the experimental value vs. our prediction as the primary evaluation metric suggested by the challenge organizers. The primary metric is defined as follows:

4 $$WAPCC=\frac{\sum _{i\mathrm{=1}}^{N}\sqrt{{n}_{i}-1}\cdot {\rho }_{i}}{\sum _{i\mathrm{=1}}^{N}\sqrt{{n}_{i}-1}}$$

where *N* = 167 is the number of the tested drug combinations, *ρ*_*i*_ is the Pearson correlation for drug combinations *i*, *n*_*i*_ is the number of cell lines that drug combination *i* is applied to.

The reported metrics are not on the final test set, and are rather cross-validation scores. All the performances we achieve should thus be considered post-hoc analyses and may appear higher than our final performance on the challenge itself.

#### Biological interpretation with feature importance

To analyze the relative predictive power of the different biological classes of features and identify potential biomarkers, we calculated how much each feature increased the accuracy of the XGBoost model (termed ‘gain’)^18^.

## Data Availability

Challenge data is available through registration via https://openinnovation.astrazeneca.com/data-library.html and it is expected to be public soon. Code is accessible via https://www.synapse.org/#!Synapse:syn5605365/wiki/394725 and https://github.com/rcelebi/dream-drugcombo.

## References

[1] Mignani S, Huber S, Tomás H, Rodrigues J, Majoral J-P (2016). Why and how have drug discovery strategies in pharma changed? What are the new mindsets?. Drug Discov. Today.

[2] Dias MH, Kitano ES, Zelanis A, Iwai LK (2016). Proteomics and drug discovery in cancer. Drug Discov. Today.

[3] Hoelder S, Clarke PA, Workman P (2012). Discovery of small molecule cancer drugs: Successes, challenges and opportunities. Mol Oncol.

[4] Lavecchia A, Cerchia C (2016). In silico methods to address polypharmacology: current status, applications and future perspectives. Drug Discov. Today.

[5] McGranahan N, Swanton C (2015). Biological and Therapeutic Impact of Intratumor Heterogeneity in Cancer Evolution. Cancer Cell.

[6] Al-Lazikani B, Banerji U, Workman P (2012). Combinatorial drug therapy for cancer in the post-genomic era. Nat Biotech.

[7] Bozic I (2013). Evolutionary dynamics of cancer in response to targeted combination therapy. eLife.

[8] Hu C-MJ, Zhang L (2012). Nanoparticle-based combination therapy toward overcoming drug resistance in cancer. Biochem. Pharmacol..

[9] Ma Y (2014). High-Dose Parenteral Ascorbate Enhanced Chemosensitivity of Ovarian Cancer and Reduced Toxicity of Chemotherapy. Science Translational Medicine.

[10] Griner LAM (2014). High-throughput combinatorial screening identifies drugs that cooperate with ibrutinib to kill activated B-cell—like diffuse large B-cell lymphoma cells. PNAS.

[11] Huang L (2014). DrugComboRanker: drug combination discovery based on target network analysis. Bioinformatics.

[12] Bansal M (2014). A community computational challenge to predict the activity of pairs of compounds. Nat Biotech.

[13] Sun, Y. *et al*. Combining genomic and network characteristics for extended capability in predicting synergistic drugs for cancer. *Nat Commun* **6**, 10.1038/ncomms9481 (2015). 10.1038/ncomms9481 PMC4598846 26412466

[14] Huang, H., Zhang, P., Qu, X. A., Sanseau, P. & Yang, L. Systematic prediction of drug combinations based on clinical side-effects. *Sci. reports* **4** (2014). 10.1038/srep07160 PMC4241517 25418113

[15] Li, X. *et al*. Prediction of synergistic anti-cancer drug combinations based on drug target network and drug induced gene expression profiles. *Artif. Intell. Medicine* (2017). 10.1016/j.artmed.2017.05.008 28583437

[16] Zhao X-M (2011). Prediction of Drug Combinations by Integrating Molecular and Pharmacological Data. PLOS Comput Biol.

[17] Menden, M. P. *et al*. Community assessment of cancer drug combination screens identifies strategies for synergy prediction. bioRxiv 200451, 10.1101/200451 (2018).

[18] Chen, T. & Guestrin, C. XGBoost: A Scalable Tree Boosting System. arXiv:1603.02754 [cs] 785–794, 10.1145/2939672.2939785 (2016).

[19] Chu W-M (2013). Tumor necrosis factor. Cancer Letters.

[20] Andrulis M (2013). Targeting the BRAF V600e mutation in multiple myeloma. Cancer Discov.

[21] Toledo LI, Murga M, Fernandez-Capetillo O (2011). Targeting ATR and Chk1 kinases for cancer treatment: A new model for new (and old) drugs. Molecular Oncology.

[22] Tseng Y-Y (2014). PVT1 dependence in cancer with MYC copy-number increase. Nature.

[23] Patane M (2013). Frequency of NFKBIA deletions is low in glioblastomas and skewed in glioblastoma neurospheres. Mol. Cancer.

[24] Ruiz N, Gronenberg LS, Kahne D, Silhavy TJ (2008). Identification of two inner-membrane proteins required for the transport of lipopolysaccharide to the outer membrane of Escherichia coli. Proc. Natl. Acad. Sci. USA.

[25] Mitrofanova A (2015). Predicting Drug Response in Human Prostate Cancer from Preclinical Analysis of *In Vivo* Mouse Models. Cell Reports.

[26] Geary N (2013). Understanding synergy. Am. J. Physiol. Endocrinol. Metab..

[27] Melville JL, Hirst JD (2007). Tmacc interpretable correlation descriptors for quantitative structure activity relationships. J. Chem. Inf. Model..

[28] O’Boyle NM (2011). Open Babel: An open chemical toolbox. Journal of Cheminformatics.

[29] Durant JL, Leland BA, Henry DR, Nourse JG (2002). Reoptimization of MDL Keys for Use in Drug Discovery. J. Chem. Inf. Comput. Sci..

[30] Finn RD (2016). The Pfam protein families database: towards a more sustainable future. Nucl. Acids Res..

[31] Sigrist CJA (2013). New and continuing developments at PROSITE. Nucleic Acids Res..

[32] Letunic I, Doerks T, Bork P (2015). SMART: recent updates, new developments and status in 2015. Nucl. Acids Res..

[33] Wilson D (2009). Superfamily sophisticated comparative genomics, data mining, visualization and phylogeny. Nucl. Acids Res..

[34] Xu K-J, Song J, Zhao X-M (2012). The drug cocktail network. BMC Syst Biol.

[35] Adamic LA, Adar E (2003). Friends and neighbors on the Web. Social Networks.

[36] Dry, J. *et al*. AstraZeneca-Sanger Drug Combination Prediction DREAM Challenge - syn4231880 (2015).

[37] Iorio F (2016). A Landscape of Pharmacogenomic Interactions in. Cancer. Cell.

[38] Langfelder P, Horvath S (2008). WGCNA: an R package for weighted correlation network analysis. BMC Bioinformatics.

[39] Langfelder P, Zhang B, Horvath S (2008). Defining clusters from a hierarchical cluster tree: the Dynamic Tree Cut package for R. Bioinformatics.

[40] Forbes SA (2015). COSMIC: exploring the world’s knowledge of somatic mutations in human cancer. Nucleic Acids Res..

[41] Seton-Rogers ST (2015). Mutant relationships. Nat Rev Cancer.

[42] Shlien A, Malkin D (2009). Copy number variations and cancer. Genome Med.

[43] Pedregosa F (2011). Scikit-learn: Machine Learning in Python. J. Mach. Learn. Res..

[44] Nik-Zainal S (2016). Landscape of somatic mutations in 560 breast cancer whole-genome sequences. Nature.

[45] Arthur JSC, Ley SC (2013). Mitogen-activated protein kinases in innate immunity. Nat Rev Immunol.

[46] Greuber EK, Smith-Pearson P, Wang J, Pendergast AM (2013). Role of ABL Family Kinases in Cancer: from Leukemia to Solid Tumors. Nat Rev Cancer.

[47] Fletcher MNC (2013). Master regulators of FGFR2 signalling and breast cancer risk. Nat Commun.

[48] Paplomata E, O’Regan R (2014). The PI3k/AKT/mTOR pathway in breast cancer: targets, trials and biomarkers. Ther Adv Med Oncol.

